# Publisher Correction: Direct observation of the dead-cone effect in quantum chromodynamics

**DOI:** 10.1038/s41586-022-05026-z

**Published:** 2022-07-07

**Authors:** ALICE Collaboration, S. Acharya, D. Adamova, A. Adler, J. Adolfsson, G. Aglieri Rinella, M. Agnello, N. Agrawal, Z. Ahammed, S. Ahmad, S. U. Ahn, I. Ahuja, Z. Akbar, A. Akindinov, M. Al-Turany, S. N. Alam, D. Aleksandrov, B. Alessandro, H. M. Alfanda, R. Alfaro Molina, B. Ali, Y. Ali, A. Alici, N. Alizadehvandchali, A. Alkin, J. Alme, T. Alt, L. Altenkamper, I. Altsybeev, M. N. Anaam, C. Andrei, D. Andreou, A. Andronic, M. Angeletti, V. Anguelov, F. Antinori, P. Antonioli, C. Anuj, N. Apadula, L. Aphecetche, H. Appelshauser, S. Arcelli, R. Arnaldi, I. C. Arsene, M. Arslandok, A. Augustinus, R. Averbeck, S. Aziz, M. D. Azmi, A. Badala, Y. W. Baek, X. Bai, R. Bailhache, Y. Bailung, R. Bala, A. Balbino, A. Baldisseri, B. Balis, M. Ball, D. Banerjee, R. Barbera, L. Barioglio, M. Barlou, G. G. Barnafoldi, L. S. Barnby, V. Barret, C. Bartels, K. Barth, E. Bartsch, F. Baruffaldi, N. Bastid, S. Basu, G. Batigne, B. Batyunya, D. Bauri, J. L. Bazo Alba, I. G. Bearden, C. Beattie, I. Belikov, A. D. C. Bell Hechavarria, F. Bellini, R. Bellwied, S. Belokurova, V. Belyaev, G. Bencedi, S. Beole, A. Bercuci, Y. Berdnikov, A. Berdnikova, L. Bergmann, M. G. Besoiu, L. Betev, P. P. Bhaduri, A. Bhasin, M. A. Bhat, B. Bhattacharjee, P. Bhattacharya, L. Bianchi, N. Bianchi, J. Bielˇcik, J. Bielˇcikova, J. Biernat, A. Bilandzic, G. Biro, S. Biswas, J. T. Blair, D. Blau, M. B. Blidaru, C. Blume, G. Boca, F. Bock, A. Bogdanov, S. Boi, J. Bok, L. Boldizsar, A. Bolozdynya, M. Bombara, P. M. Bond, G. Bonomi, H. Borel, A. Borissov, H. Bossi, E. Botta, L. Bratrud, P. Braun-Munzinger, M. Bregant, M. Broz, G. E. Bruno, M. D. Buckland, D. Budnikov, H. Buesching, S. Bufalino, O. Bugnon, P. Buhler, Z. Buthelezi, J. B. Butt, S. A. Bysiak, D. Caffarri, M. Cai, H. Caines, A. Caliva, E. Calvo Villar, J. M. M. Camacho, R. S. Camacho, P. Camerini, F. D. M. Canedo, F. Carnesecchi, R. Caron, J. Castillo Castellanos, E. A. R. Casula, F. Catalano, C. Ceballos Sanchez, P. Chakraborty, S. Chandra, S. Chapeland, M. Chartier, S. Chattopadhyay, S. Chattopadhyay, A. Chauvin, T. G. Chavez, C. Cheshkov, B. Cheynis, V. Chibante Barroso, D. D. Chinellato, S. Cho, P. Chochula, P. Christakoglou, C. H. Christensen, P. Christiansen, T. Chujo, C. Cicalo, L. Cifarelli, F. Cindolo, M. R. Ciupek, G. Clai, J. Cleymans, F. Colamaria, J. S. Colburn, D. Colella, A. Collu, M. Colocci, M. Concas, G. Conesa Balbastre, Z. Conesa del Valle, G. Contin, J. G. Contreras, M. L. Coquet, T. M. Cormier, P. Cortese, M. R. Cosentino, F. Costa, S. Costanza, P. Crochet, R. Cruz-Torres, E. Cuautle, P. Cui, L. Cunqueiro, A. Dainese, F. P. A. Damas, M. C. Danisch, A. Danu, I. Das, P. Das, P. Das, S. Das, S. Dash, S. De, A. De Caro, G. de Cataldo, L. De Cilladi, J. de Cuveland, A. De Falco, D. De Gruttola, N. De Marco, C. De Martin, S. De Pasquale, S. Deb, H. F. Degenhardt, K. R. Deja, L. Dello Stritto, S. Delsanto, W. Deng, P. Dhankher, D. Di Bari, A. Di Mauro, R. A. Diaz, T. Dietel, Y. Ding, R. Divia, D. U. Dixit, O. Djuvsland, U. Dmitrieva, J. Do, A. Dobrin, B. Donigus, O. Dordic, A. K. Dubey, A. Dubla, S. Dudi, M. Dukhishyam, P. Dupieux, N. Dzalaiova, T. M. Eder, R. J. Ehlers, V. N. Eikeland, F. Eisenhut, D. Elia, B. Erazmus, F. Ercolessi, F. Erhardt, A. Erokhin, M. R. Ersdal, B. Espagnon, G. Eulisse, D. Evans, S. Evdokimov, L. Fabbietti, M. Faggin, J. Faivre, F. Fan, A. Fantoni, M. Fasel, P. Fecchio, A. Feliciello, G. Feofilov, A. Fernandez Tellez, A. Ferrero, A. Ferretti, V. J. G. Feuillard, J. Figiel, S. Filchagin, D. Finogeev, F. M. Fionda, G. Fiorenza, F. Flor, A. N. Flores, S. Foertsch, P. Foka, S. Fokin, E. Fragiacomo, E. Frajna, U. Fuchs, N. Funicello, C. Furget, A. Furs, J. J. Gaardhoje, M. Gagliardi, A. M. Gago, A. Gal, C. D. Galvan, P. Ganoti, C. Garabatos, J. R. A. Garcia, E. Garcia-Solis, K. Garg, C. Gargiulo, A. Garibli, K. Garner, P. Gasik, E. F. Gauger, A. Gautam, M. B. Gay Ducati, M. Germain, P. Ghosh, S. K. Ghosh, M. Giacalone, P. Gianotti, P. Giubellino, P. Giubilato, A. M. C. Glaenzer, P. Glassel, D. J. Q. Goh, V. Gonzalez, L. H. Gonzalez-Trueba, S. Gorbunov, M. Gorgon, L. Gorlich, S. Gotovac, V. Grabski, L. K. Graczykowski, L. Greiner, A. Grelli, C. Grigoras, V. Grigoriev, A. Grigoryan, S. Grigoryan, O. S. Groettvik, F. Grosa, J. F. Grosse-Oetringhaus, R. Grosso, G. G. Guardiano, R. Guernane, M. Guilbaud, K. Gulbrandsen, T. Gunji, A. Gupta, R. Gupta, S. P. Guzman, L. Gyulai, M. K. Habib, C. Hadjidakis, G. Halimoglu, H. Hamagaki, G. Hamar, M. Hamid, R. Hannigan, M. R. Haque, A. Harlenderova, J. W. Harris, A. Harton, J. A. Hasenbichler, H. Hassan, D. Hatzifotiadou, P. Hauer, L. B. Havener, S. Hayashi, S. T. Heckel, E. Hellbar, H. Helstrup, T. Herman, E. G. Hernandez, G. Herrera Corral, F. Herrmann, K. F. Hetland, H. Hillemanns, C. Hills, B. Hippolyte, B. Hofman, B. Hohlweger, J. Honermann, G. H. Hong, D. Horak, S. Hornung, A. Horzyk, R. Hosokawa, P. Hristov, C. Hughes, P. Huhn, T. J. Humanic, H. Hushnud, L. A. Husova, A. Hutson, D. Hutter, J. P. Iddon, R. Ilkaev, H. Ilyas, M. Inaba, G. M. Innocenti, M. Ippolitov, A. Isakov, M. S. Islam, M. Ivanov, V. Ivanov, V. Izucheev, M. Jablonski, B. Jacak, N. Jacazio, P. M. Jacobs, S. Jadlovska, J. Jadlovsky, S. Jaelani, C. Jahnke, M. J. Jakubowska, A. Jalotra, M. A. Janik, T. Janson, M. Jercic, O. Jevons, F. Jonas, P. G. Jones, J. M. Jowett, J. Jung, M. Jung, A. Junique, A. Jusko, J. Kaewjai, P. Kalinak, A. Kalweit, V. Kaplin, S. Kar, A. Karasu Uysal, D. Karatovic, O. Karavichev, T. Karavicheva, P. Karczmarczyk, E. Karpechev, A. Kazantsev, U. Kebschull, R. Keidel, D. L. D. Keijdener, M. Keil, B. Ketzer, Z. Khabanova, A. M. Khan, S. Khan, A. Khanzadeev, Y. Kharlov, A. Khatun, A. Khuntia, B. Kileng, B. Kim, C. Kim, D. Kim, D. J. Kim, E. J. Kim, J. Kim, J. S. Kim, J. Kim, J. Kim, M. Kim, S. Kim, T. Kim, S. Kirsch, I. Kisel, S. Kiselev, A. Kisiel, J. P. Kitowski, J. L. Klay, J. Klein, S. Klein, C. Klein-Bosing, M. Kleiner, T. Klemenz, A. Kluge, A. G. Knospe, C. Kobdaj, M. K. Kohler, T. Kollegger, A. Kondratyev, N. Kondratyeva, E. Kondratyuk, J. Konig, S. A. Konigstorfer, P. J. Konopka, G. Kornakov, S. D. Koryciak, L. Koska, A. Kotliarov, O. Kovalenko, V. Kovalenko, M. Kowalski, I. Kralik, A. Kravˇcakova, L. Kreis, M. Krivda, F. Krizek, K. Krizkova Gajdosova, M. Kroesen, M. Kruger, E. Kryshen, M. Krzewicki, V. Kuˇcera, C. Kuhn, P. G. Kuijer, T. Kumaoka, D. Kumar, L. Kumar, N. Kumar, S. Kundu, P. Kurashvili, A. Kurepin, A. B. Kurepin, A. Kuryakin, S. Kushpil, J. Kvapil, M. J. Kweon, J. Y. Kwon, Y. Kwon, S. L. La Pointe, P. La Rocca, Y. S. Lai, A. Lakrathok, M. Lamanna, R. Langoy, K. Lapidus, P. Larionov, E. Laudi, L. Lautner, R. Lavicka, T. Lazareva, R. Lea, J. Lehrbach, R. C. Lemmon, I. Leon Monzon, E. D. Lesser, M. Lettrich, P. Levai, X. Li, X. L. Li, J. Lien, R. Lietava, B. Lim, S. H. Lim, V. Lindenstruth, A. Lindner, C. Lippmann, A. Liu, J. Liu, I. M. Lofnes, V. Loginov, C. Loizides, P. Loncar, J. A. Lopez, X. Lopez, E. Lopez Torres, J. R. Luhder, M. Lunardon, G. Luparello, Y. G. Ma, A. Maevskaya, M. Mager, T. Mahmoud, A. Maire, M. Malaev, N. M. Malik, Q. W. Malik, L. Malinina, D. Mal’Kevich, N. Mallick, P. Malzacher, G. Mandaglio, V. Manko, F. Manso, V. Manzari, Y. Mao, J. Mareš, G. V. Margagliotti, A. Margotti, A. Marin, C. Markert, M. Marquard, N. A. Martin, P. Martinengo, J. L. Martinez, M. I. Martinez, G. Martinez Garcia, S. Masciocchi, M. Masera, A. Masoni, L. Massacrier, A. Mastroserio, A. M. Mathis, O. Matonoha, P. F. T. Matuoka, A. Matyja, C. Mayer, A. L. Mazuecos, F. Mazzaschi, M. Mazzilli, M. A. Mazzoni, J. E. Mdhluli, A. F. Mechler, F. Meddi, Y. Melikyan, A. Menchaca-Rocha, E. Meninno, A. S. Menon, M. Meres, S. Mhlanga, Y. Miake, L. Micheletti, L. C. Migliorin, D. L. Mihaylov, K. Mikhaylov, A. N. Mishra, D. Mi´skowiec, A. Modak, A. P. Mohanty, B. Mohanty, M. Mohisin Khan, Z. Moravcova, C. Mordasini, D. A. Moreira De Godoy, L. A. P. Moreno, I. Morozov, A. Morsch, T. Mrnjavac, V. Muccifora, E. Mudnic, D. Muhlheim, S. Muhuri, J. D. Mulligan, A. Mulliri, M. G. Munhoz, R. H. Munzer, H. Murakami, S. Murray, L. Musa, J. Musinsky, J. W. Myrcha, B. Naik, R. Nair, B. K. Nandi, R. Nania, E. Nappi, M. U. Naru, A. F. Nassirpour, A. Nath, C. Nattrass, A. Neagu, L. Nellen, S. V. Nesbo, G. Neskovic, D. Nesterov, B. S. Nielsen, S. Nikolaev, S. Nikulin, V. Nikulin, F. Noferini, S. Noh, P. Nomokonov, J. Norman, N. Novitzky, P. Nowakowski, A. Nyanin, J. Nystrand, M. Ogino, A. Ohlson, V. A. Okorokov, J. Oleniacz, A. C. Oliveira Da Silva, M. H. Oliver, A. Onnerstad, C. Oppedisano, A. Ortiz Velasquez, T. Osako, A. Oskarsson, J. Otwinowski, K. Oyama, Y. Pachmayer, S. Padhan, D. Pagano, G. Pai´c, A. Palasciano, J. Pan, S. Panebianco, P. Pareek, J. Park, J. E. Parkkila, S. P. Pathak, R. N. Patra, B. Paul, J. Pazzini, H. Pei, T. Peitzmann, X. Peng, L. G. Pereira, H. Pereira Da Costa, D. Peresunko, G. M. Perez, S. Perrin, Y. Pestov, V. Petráček, M. Petrovici, R. P. Pezzi, S. Piano, M. Pikna, P. Pillot, O. Pinazza, L. Pinsky, C. Pinto, S. Pisano, M. Płoskoń, M. Planinic, F. Pliquett, M. G. Poghosyan, B. Polichtchouk, S. Politano, N. Poljak, A. Pop, S. Porteboeuf-Houssais, J. Porter, V. Pozdniakov, S. K. Prasad, R. Preghenella, F. Prino, C. A. Pruneau, I. Pshenichnov, M. Puccio, S. Qiu, L. Quaglia, R. E. Quishpe, S. Ragoni, A. Rakotozafindrabe, L. Ramello, F. Rami, S. A. R. Ramirez, A. G. T. Ramos, T. A. Rancien, R. Raniwala, S. Raniwala, S. S. Rasanen, R. Rath, I. Ravasenga, K. F. Read, A. R. Redelbach, K. Redlich, A. Rehman, P. Reichelt, F. Reidt, H. A. Reme-ness, R. Renfordt, Z. Rescakova, K. Reygers, A. Riabov, V. Riabov, T. Richert, M. Richter, W. Riegler, F. Riggi, C. Ristea, S. P. Rode, M. Rodriguez Cahuantzi, K. Roed, R. Rogalev, E. Rogochaya, T. S. Rogoschinski, D. Rohr, D. Rohrich, P. F. Rojas, P. S. Rokita, F. Ronchetti, A. Rosano, E. D. Rosas, A. Rossi, A. Rotondi, A. Roy, P. Roy, S. Roy, N. Rubini, O. V. Rueda, R. Rui, B. Rumyantsev, P. G. Russek, A. Rustamov, E. Ryabinkin, Y. Ryabov, A. Rybicki, H. Rytkonen, W. Rzesa, O. A. M. Saarimaki, R. Sadek, S. Sadovsky, J. Saetre, K. Šafařík, S. K. Saha, S. Saha, B. Sahoo, P. Sahoo, R. Sahoo, S. Sahoo, D. Sahu, P. K. Sahu, J. Saini, S. Sakai, S. Sambyal, V. Samsonov, D. Sarkar, N. Sarkar, P. Sarma, V. M. Sarti, M. H. P. Sas, J. Schambach, H. S. Scheid, C. Schiaua, R. Schicker, A. Schmah, C. Schmidt, H. R. Schmidt, M. O. Schmidt, M. Schmidt, N. V. Schmidt, A. R. Schmier, R. Schotter, J. Schukraft, Y. Schutz, K. Schwarz, K. Schweda, G. Scioli, E. Scomparin, J. E. Seger, Y. Sekiguchi, D. Sekihata, I. Selyuzhenkov, S. Senyukov, J. J. Seo, D. Serebryakov, L. Šerkšnytė, A. Sevcenco, T. J. Shaba, A. Shabanov, A. Shabetai, R. Shahoyan, W. Shaikh, A. Shangaraev, A. Sharma, H. Sharma, M. Sharma, N. Sharma, S. Sharma, U. Sharma, O. Sheibani, K. Shigaki, M. Shimomura, S. Shirinkin, Q. Shou, Y. Sibiriak, S. Siddhanta, T. Siemiarczuk, T. F. Silva, D. Silvermyr, G. Simonetti, B. Singh, R. Singh, R. Singh, R. Singh, V. K. Singh, V. Singhal, T. Sinha, B. Sitar, M. Sitta, T. B. Skaali, G. Skorodumovs, M. Slupecki, N. Smirnov, R. J. M. Snellings, C. Soncco, J. Song, A. Songmoolnak, F. Soramel, S. Sorensen, I. Sputowska, J. Stachel, I. Stan, P. J. Steffanic, S. F. Stiefelmaier, D. Stocco, I. Storehaug, M. M. Storetvedt, C. P. Stylianidis, A. A. P. Suaide, T. Sugitate, C. Suire, M. Suljic, R. Sultanov, M. Šumbera, V. Sumberia, S. Sumowidagdo, S. Swain, A. Szabo, I. Szarka, U. Tabassam, S. F. Taghavi, G. Taillepied, J. Takahashi, G. J. Tambave, S. Tang, Z. Tang, M. Tarhini, M. G. Tarzila, A. Tauro, G. Tejeda Munoz, A. Telesca, L. Terlizzi, C. Terrevoli, G. Tersimonov, S. Thakur, D. Thomas, R. Tieulent, A. Tikhonov, A. R. Timmins, M. Tkacik, A. Toia, N. Topilskaya, M. Toppi, F. Torales-Acosta, T. Tork, S. R. Torres, A. Trifiro, S. Tripathy, T. Tripathy, S. Trogolo, G. Trombetta, V. Trubnikov, W. H. Trzaska, T. P. Trzcinski, B. A. Trzeciak, A. Tumkin, R. Turrisi, T. S. Tveter, K. Ullaland, A. Uras, M. Urioni, G. L. Usai, M. Vala, N. Valle, S. Vallero, N. van der Kolk, L. V. R. van Doremalen, M. van Leeuwen, P. Vande Vyvre, D. Varga, Z. Varga, M. Varga-Kofarago, A. Vargas, M. Vasileiou, A. Vasiliev, O. Vazquez Doce, V. Vechernin, E. Vercellin, S. Vergara Limon, L. Vermunt, R. Vertesi, M. Verweij, L. Vickovic, Z. Vilakazi, O. Villalobos Baillie, G. Vino, A. Vinogradov, T. Virgili, V. Vislavicius, A. Vodopyanov, B. Volkel, M. A. Volkl, K. Voloshin, S. A. Voloshin, G. Volpe, B. von Haller, I. Vorobyev, D. Voscek, N. Vozniuk, J. Vrlakova, B. Wagner, C. Wang, D. Wang, M. Weber, R. J. G. V. Weelden, A. Wegrzynek, S. C. Wenzel, J. P. Wessels, J. Wiechula, J. Wikne, G. Wilk, J. Wilkinson, G. A. Willems, B. Windelband, M. Winn, W. E. Witt, J. R. Wright, W. Wu, Y. Wu, R. Xu, S. Yalcin, Y. Yamaguchi, K. Yamakawa, S. Yang, S. Yano, Z. Yin, H. Yokoyama, I.-K. Yoo, J. H. Yoon, S. Yuan, A. Yuncu, V. Zaccolo, A. Zaman, C. Zampolli, H. J. C. Zanoli, N. Zardoshti, A. Zarochentsev, P. Zavada, N. Zaviyalov, H. Zbroszczyk, M. Zhalov, S. Zhang, X. Zhang, Y. Zhang, V. Zherebchevskii, Y. Zhi, N. Zhigareva, D. Zhou, Y. Zhou, J. Zhu, Y. Zhu, A. Zichichi, G. Zinovjev, N. Zurlo

**Affiliations:** 1 Variable Energy Cyclotron Centre, Homi Bhabha National Institute, Kolkata, India; 2 Nuclear Physics Institute of the Czech Academy of Sciences, Řežu Prahy, Czech Republic; 3 Institut fur Informatik, Fachbereich Informatik und Mathematik, Johann-Wolfgang-Goethe Universitat Frankfurt, Frankfurt, Germany; 4 Department of Physics, Division of Particle Physics, Lund University, Lund, Sweden; 5 European Organization for Nuclear Research (CERN), Geneva, Switzerland; 6 Dipartimento DISAT del Politecnico and Sezione INFN, Turin, Italy; 7 INFN, Sezione di Bologna, Bologna, Italy; 8 Department of Physics, Aligarh Muslim University, Aligarh, India; 9 Korea Institute of Science and Technology Information, Daejeon, Republic of Korea; 10 Faculty of Science, P.J. Šafarik University, Košice, Slovakia; 11 Indonesian Institute of Sciences, Jakarta, Indonesia; 12 NRC ≫Kurchatov ≫ Institute – ITEP, Moscow, Russia; 13 Research Division and ExtreMe Matter Institute EMMI, GSI Helmholtzzentrum fur Schwerionenforschung GmbH, Darmstadt, Germany; 14 Fudan University, Shanghai, China; 15 National Research Centre Kurchatov Institute, Moscow, Russia; 16 INFN, Sezione di Torino, Turin, Italy; 17 Central China Normal University, Wuhan, China; 18 Instituto de Fisica, Universidad Nacional Autonoma de Mexico, Mexico City, Mexico; 19 COMSATS University Islamabad, Islamabad, Pakistan; 20 Dipartimento di Fisica e Astronomia dell’Universita and Sezione INFN, Bologna, Italy; 21 University of Houston, Houston, TX USA; 22 Department of Physics and Technology, University of Bergen, Bergen, Norway; 23 Institut fur Kernphysik, Johann Wolfgang Goethe-Universitat Frankfurt, Frankfurt, Germany; 24 St. Petersburg State University, St. Petersburg, Russia; 25 Horia Hulubei National Institute of Physics and Nuclear Engineering, Bucharest, Romania; 26 Nikhef, National Institute for Subatomic Physics, Amsterdam, the Netherlands; 27 Institut fur Kernphysik, Westfalische Wilhelms-Universitat Munster, Munster, Germany; 28 Physikalisches Institut, Ruprecht-Karls-Universitat Heidelberg, Heidelberg, Germany; 29 INFN, Sezione di Padova, Padova, Italy; 30 Lawrence Berkeley National Laboratory, Berkeley, CA USA; 31 SUBATECH, IMT Atlantique, Universite de Nantes, CNRS-IN2P3, Nantes, France; 32 Department of Physics, University of Oslo, Oslo, Norway; 33 Yale University, New Haven, CT USA; 34 Laboratoire de Physique des 2 Infinis, Irene Joliot-Curie, Orsay, France; 35 INFN, Sezione di Catania, Catania, Italy; 36 Gangneung-Wonju National University, Gangneung, Republic of Korea; 37 University of Science and Technology of China, Hefei, China; 38 Indian Institute of Technology Indore, Indore, India; 39 Physics Department, University of Jammu, Jammu, India; 40 Department de Physique Nucleaire (DPhN), Universite Paris-Saclay Centre d’Etudes de Saclay (CEA), IRFU, Saclay, France; 41 AGH University of Science and Technology, Cracow, Poland; 42 Helmholtz-Institut fur Strahlen- und Kernphysik, Rheinische Friedrich-Wilhelms-Universitat Bonn, Bonn, Germany; 43 Bose Institute, Department of Physics and Centre for Astroparticle Physics and Space Science (CAPSS), Kolkata, India; 44 Dipartimento di Fisica e Astronomia dell’Universita and Sezione INFN, Catania, Italy; 45 Physik Department, Technische Universitat Munchen, Munich, Germany; 46 Dipartimento di Fisica dell’Universita and Sezione INFN, Turin, Italy; 47 Department of Physics, School of Science, National and Kapodistrian University of Athens,, Athens, Greece; 48 Wigner Research Centre for Physics, Budapest, Hungary; 49 Nuclear Physics Group, STFC Daresbury Laboratory, Daresbury, UK; 50 Universite Clermont Auvergne, CNRS/IN2P3, LPC, Clermont-Ferrand, France; 51 University of Liverpool, Liverpool, UK; 52 Dipartimento di Fisica e Astronomia dell’Universita and Sezione INFN, Padova, Italy; 53 Joint Institute for Nuclear Research (JINR), Dubna, Russia; 54 Indian Institute of Technology Bombay (IIT), Mumbai, India; 55 Seccion Fisica, Departamento de Ciencias, Pontificia Universidad Catolica del Peru, Lima, Peru; 56 Niels Bohr Institute, University of Copenhagen, Copenhagen, Denmark; 57 Universite de Strasbourg, CNRS, IPHC UMR 7178, Strasbourg, France; 58 NRNU Moscow Engineering Physics Institute, Moscow, Russia; 59 Instituto de Ciencias Nucleares, Universidad Nacional Autonoma de Mexico, Mexico City, Mexico; 60 Petersburg Nuclear Physics Institute, Gatchina, Russia; 61 Institute of Space Science (ISS), Bucharest, Romania; 62 Department of Physics, Gauhati University, Guwahati, India; 63 Dipartimento di Fisica dell’Universita and Sezione INFN, Cagliari, Italy; 64 INFN, Laboratori Nazionali di Frascati, Frascati, Italy; 65 Faculty of Nuclear Sciences and Physical Engineering, Czech Technical University in Prague, Prague, Czech Republic; 66 The Henryk Niewodniczanski Institute of Nuclear Physics, Polish Academy of Sciences, Cracow, Poland; 67 The University of Texas at Austin, Austin, TX USA; 68 Dipartimento di Fisica e Nucleare e Teorica, Universita di Pavia, Pavia, Italy; 69 INFN, Sezione di Pavia, Pavia, Italy; 70 Oak Ridge National Laboratory, Oak Ridge, TN USA; 71 Inha University, Incheon, Republic of Korea; 72 Universita di Brescia, Brescia, Italy; 73 Moscow Institute for Physics and Technology, Moscow, Russia; 74 Universidade de Sao Paulo (USP), Sao Paulo, Brazil; 75 Politecnico di Bari and Sezione INFN, Bari, Italy; 76 Dipartimento Interateneo di Fisica ‘M. Merlin’ and Sezione INFN, Bari, Italy; 77 Russian Federal Nuclear Center (VNIIEF), Sarov, Russia; 78 Stefan Meyer Institut fur Subatomare Physik (SMI), Vienna, Austria; 79 iThemba LABS, National Research Foundation, Somerset West, South Africa; 80 University of the Witwatersrand, Johannesburg, South Africa; 81 Universidad Autonoma de Sinaloa, Culiacan, Mexico; 82 High Energy Physics Group, Universidad Autonoma de Puebla, Puebla, Mexico; 83 Dipartimento di Fisica dell’Universita and Sezione INFN, Trieste, Italy; 84 Saha Institute of Nuclear Physics, Homi Bhabha National Institute, Kolkata, India; 85 Universite de Lyon, CNRS/IN2P3, Institut de Physique des 2 Infinis de Lyon, Lyon, France; 86 Universidade Estadual de Campinas (UNICAMP), Campinas, Brazil; 87 University of Tsukuba, Tsukuba, Japan; 88 INFN, Sezione di Cagliari, Cagliari, Italy; 89 University of Cape Town, Cape Town, South Africa; 90 INFN, Sezione di Bari, Bari, Italy; 91 School of Physics and Astronomy, University of Birmingham, Birmingham, UK; 92 Laboratoire de Physique Subatomique et de Cosmologie, Universite Grenoble-Alpes, CNRS-IN2P3, Grenoble, France; 93 Dipartimento di Scienze e Innovazione Tecnologica dell’Universita del Piemonte Orientale and INFN Sezione di Torino, Alessandria, Italy; 94 Universidade Federal do ABC, Santo Andre, Brazil; 95 National Institute of Science Education and Research, Homi Bhabha National Institute, Jatni, India; 96 Dipartimento di Fisica ‘E.R. Caianiello’ dell’Universita and Gruppo Collegato INFN, Salerno, Italy; 97 Frankfurt Institute for Advanced Studies, Johann Wolfgang Goethe-Universitat Frankfurt, Frankfurt, Germany; 98 Warsaw University of Technology, Warsaw, Poland; 99 Department of Physics, University of California, Berkeley, CA USA; 100 Centro de Aplicaciones Tecnologicas y Desarrollo Nuclear (CEADEN), Havana, Cuba; 101 Institute for Nuclear Research, Academy of Sciences, Moscow, Russia; 102 Physics Department, Panjab University, Chandigarh, India; 103 Comenius University Bratislava, Faculty of Mathematics, Physics and Informatics, Bratislava, Slovakia; 104 Physics Department, Faculty of Science, University of Zagreb, Zagreb, Croatia; 105 NRC Kurchatov Institute IHEP, Protvino, Russia; 106 INFN, Sezione di Trieste, Trieste, Italy; 107 Chicago State University, Chicago, IL USA; 108 National Nuclear Research Center, Baku, Azerbaijan; 109 University of Kansas, Lawrence, KS USA; 110 Instituto de Fisica, Universidade Federal do Rio Grande do Sul (UFRGS), Porto Alegre, Brazil; 111 Nagasaki Institute of Applied Science, Nagasaki, Japan; 112 Wayne State University, Detroit, MI USA; 113 Faculty of Electrical Engineering, Mechanical Engineering and Naval Architecture, University of Split, Split, Croatia; 114 Institute for Gravitational and Subatomic Physics (GRASP), Utrecht University/Nikhef, Utrecht, the Netherlands; 115 A.I. Alikhanyan National Science Laboratory (Yerevan Physics Institute) Foundation, Yerevan, Armenia; 116 University of Tokyo, Tokyo, Japan; 117 Faculty of Engineering and Science, Western Norway University of Applied Sciences, Bergen, Norway; 118 Centro de Investigacion y de Estudios Avanzados (CINVESTAV), Mexico City and Merida, Mexico; 119 Yonsei University, Seoul, Republic of Korea; 120 Creighton University, Omaha, NE USA; 121 University of Tennessee, Knoxville, TN USA; 122 Ohio State University, Columbus, OH USA; 123 Technical University of Košice, Košice, Slovakia; 124 Suranaree University of Technology, Nakhon Ratchasima, Thailand; 125 Institute of Experimental Physics, Slovak Academy of Sciences, Košice, Slovakia; 126 KTO Karatay University, Konya, Turkey; 127 Hochschule Worms, Zentrum fur Technologietransfer und Telekommunikation (ZTT), Worms, Germany; 128 Department of Physics, Pusan National University, Pusan, Republic of Korea; 129 University of Jyvaskyla, Jyvaskyla, Finland; 130 Jeonbuk National University, Jeonju, Republic of Korea; 131 Department of Physics, Sejong University, Seoul, Republic of Korea; 132 California Polytechnic State University, San Luis Obispo, CA USA; 133 National Centre for Nuclear Research, Warsaw, Poland; 134 University of South-Eastern Norway, Tonsberg, Norway; 135 China Institute of Atomic Energy, Beijing, China; 136 Dipartimento di Scienze MIFT, Universita di Messina, Messina, Italy; 137 Institute of Physics of the Czech Academy of Sciences, Prague, Czech Republic; 138 Universita degli Studi di Foggia, Foggia, Italy; 139 INFN, Sezione di Roma, Rome, Italy; 140 Dipartimento di Fisica dell’Universita ‘La Sapienza’ and Sezione INFN, Rome, Italy; 141 Chungbuk National University, Cheongju, Republic of Korea; 142 Hiroshima University, Hiroshima, Japan; 143 Budker Institute for Nuclear Physics, Novosibirsk, Russia; 144 Physics Department, University of Rajasthan, Jaipur, India; 145 Helsinki Institute of Physics (HIP), Helsinki, Finland; 146 Institute of Physics, Homi Bhabha National Institute, Bhubaneswar, India; 147 Physikalisches Institut, Eberhard-Karls-Universitat Tubingen, Tubingen, Germany; 148 Nara Women’s University (NWU), Nara, Japan; 149 Bogolyubov Institute for Theoretical Physics, National Academy of Sciences of Ukraine, Kiev, Ukraine; 150 Present Address: Italian National Agency for New Technologies, Energy and Sustainable Economic Development, (ENEA), Bologna, Italy; 151 Present Address: Dipartimento DET del Politecnico di Torino, Turin, Italy; 152 Present Address: D.V. Skobeltsyn Institute of Nuclear Physics, M.V. Lomonosov Moscow State University, Moscow, Russia; 153 Present Address: Institute of Theoretical Physics, University of Wroclaw, Wroclaw, Poland

**Keywords:** Experimental nuclear physics, Experimental particle physics

Correction to: *Nature* 10.1038/s41586-022-04572-w Published online 18 May 2022

In the version of this article initially published, there was a typographical error in the first sentence following the “Exposing the dead cone” heading, now reading, “The measurements of *R(θ)*, in the three radiator (charm-quark) energy intervals 5 < *E*_Radiator_ < 10 GeV, 10 < *E*_Radiator_ < 20 GeV and 20 < *E*_Radiator_ < 35 GeV…,” where “35 GeV” initially appeared as “3 GeV.” The error has been corrected in the HTML and PDF versions of the article.

